# “Changeons les Règles!” development and feasibility testing of an encounter decision aid for menstrual management in adolescents and young adults with developmental disabilities

**DOI:** 10.1016/j.pecinn.2025.100430

**Published:** 2025-09-13

**Authors:** Dehlia Moussaoui, Victoria L. Crofts, Anne-Chantal Héritier-Barras, Thomas Agoritsas, Arnaud Merglen, Michal Yaron

**Affiliations:** a Division of General Pediatrics, Department of Pediatrics, Obstetrics and Gynecology, Geneva University Hospitals and Faculty of Medicine, University of Geneva, Geneva, Switzerland; b Division of Gynecology, Department of Pediatrics, Obstetrics and Gynecology, Geneva University Hospitals and Faculty of Medicine, University of Geneva, Geneva, Switzerland; c Programme Handicap, Medical and Quality Directorate, Geneva University Hospitals, Geneva, Switzerland; d Division of General Internal Medicine, Department of Medicine, Geneva University Hospitals, Geneva, Switzerland; e Department of Health Research Methods, Evidence, and Impact, McMaster University, Hamilton, Ontario, Canada; f MAGIC Evidence Ecosystem Foundation, Oslo, Norway

**Keywords:** Menstrual management, Disability, Adolescents, Dysmenorrhea, Heavy menstrual bleeding, Decision aid, Shared decision making

## Abstract

**Objectives:**

To report the developing process and acceptability testing of a decision aid designed for adolescents and young adults with developmental disabilities, focusing on treatment options for menstrual management.

**Methods:**

We developed a paper-based encounter decision aid to support shared decision-making about treatment options for menstrual management for adolescents and young adults with developmental disabilities. This tool was designed to be both evidence-based and user-centered. We conducted a feasibility study to assess its acceptability

**Results:**

The decision aid was used during consultations with 18 adolescents and young adults with developmental disabilities and their caregivers. Participants reported high levels of acceptability and found the tool helpful in facilitating decision-making. They particularly valued the ability to compare treatment options side by side. The tool also promoted meaningful conversations between patients and clinicians

**Conclusions:**

The decision aid was well-accepted and successfully facilitated the discussion about menstrual management options between patients and clinicians. Further research is needed to evaluate its long-term impact on decision making outcomes and patient satisfaction

**Innovation:**

This innovative tool may support shared decision-making for adolescents and young adults with developmental disabilities and their caregivers, and provide additional insight on how to engage individuals with developmental disabilities in healthcare decisions.

## Highlights

• A decision aid was created to support shared decision-making about menstrual management.
• A feasibility study showed high levels of acceptability among adolescents with disability.
• The innovative tool facilitated comparison of treatment options.
• The use of the decision aid was associated with a reduction in decisional conflict.
• It provides new insight on how to engage people with disability in decision making.

## 1 Background

Menstrual disorders, such as dysmenorrhea and heavy menstrual bleeding, are highly prevalent among adolescents and young adults (AYAs) and are associated with reduced quality of life and increased school absenteeism [1]. Developmental disabilities (DD) represent a group of lifelong conditions characterized by limitations in intellectual functioning, adaptive behavior, and/or motor development, with onset before the age of 22 [2,3]. AYAs with DD experience menstrual issues similar to their peers, but face additional challenges related to their underlying condition. These include mood and behavior disturbances, difficulties managing menstrual hygiene, and catamenial seizures [4,5]. Furthermore, they are more vulnerable to sexual abuse and are at higher risk of unwanted pregnancies compared to their peers [6,7].

Families and caregivers of AYAs with DD often express concerns about puberty [8,9]. They may seek guidance for menstrual management, which involves the use of non-hormonal and hormonal medications to relieve menstrual symptoms and, in some cases, to achieve amenorrhea [10,11]. Although AYAs with DD have access to the same treatment options for menstrual management as the general population, unique aspects of their medical conditions [10,12] and ethical considerations [13] make decision-making particularly complex and challenging. AYAs with DD and their caregivers often require additional decisional support to navigate these options.

Decision aids are educational tools designed to support individuals in making informed healthcare decisions [14,15]. These tools provide evidence-based information and promote patient-centered care by fostering patient autonomy and engagement [15]. The primary goal of decision aids is to facilitate shared decision-making – a collaborative process in which patients and clinicians make decisions together about treatment or intervention, using the best available evidence and considering patient's values and preferences and practical considerations [[16], [17], [18], [19]].

Research on engaging individuals with DD in decision-making is limited, and few decision aids have been specifically developed for this population [[20], [21], [22]]. Existing decision aids have shown potential to improve shared decision-making in vulnerable populations [20], yet no decision aid currently exists for menstrual management in individuals with DD.

This manuscript outlines the developing process and acceptability testing of an encounter decision aid designed for AYAs with DD and their caregivers, focusing on treatment options for menstrual management.

## 2 Methods

### 2.1 Development of the encounter decision aid

The encounter decision aid (EDA) titled “Changeons les règles !” (« Let's change the rules! »), plays on the double meaning of the French word « règles », which refers to both rules and menstruation. The EDA was designed to facilitate the participation of AYAs with DD and their caregivers in the decision-making process for menstrual management, promoting autonomy and engagement.

We developed the EDA in alignment with the latest International Patient Decision Aids Standards (IPDAS) and the Ottawa Decision Support Framework [23,24]. The content was informed by a systematic review on menstrual management in AYAs with DD conducted by our team [25], professional societies guidelines [11,[26], [27], [28]], and stakeholder input.

To create the initial prototype, we assembled a team comprising: AYAs with experience using hormonal treatment for menstrual management, clinicians (pediatricians, gynecologists, and neurologists), a nurse specialized in pediatrics, a young adult with developmental disability, and a graphic designer. We also sought additional feedback from individuals with DD and their caregivers, a specialized nurse with lived experience of sensory disability, and an inclusivity proofreader to ensure accessibility and inclusivity.

The EDA was inspired by the pioneering work of Montori and colleagues at Mayo Clinic Knowledge and Evaluation Research Unit. Their approach emphasizes user-centred tools addressing practical issues and key outcomes relevant to patients **[****29****]**. Unlike traditional decision aids, which are often used independently by patients, Montori's methods aims to increase patient participation and ensure tailored, patient-specific information delivery during the clinical encounter **[****30****]**. This innovative approach has been used to develop several EDA covering topics such as antidepressants, statins, medications for type 2 diabetes, chest pain, atrial fibrillation, head CT and osteoporosis **[**[31], [32], [33], [34], [35], [36], [37]**]**. These tools have been demonstrated in randomized controlled trials to be effective in improving shared decision-making **[**[31], [32], [33], [34], [35], [36], [37]**]**.

**Fig. 1:**
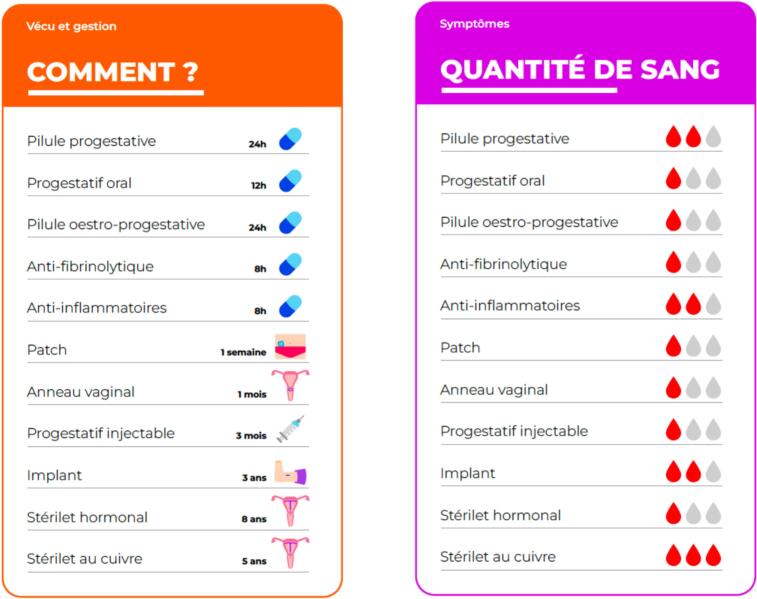
example of two cards from the encounter decision aid.

Our paper-based EDA consists of 11 cards, each addressing a single practical issue (e.g. pain, bleeding, mood changes, contraception) from a practical perspective (Fig. 1). Each card compares the available treatment options based on the specific issue it addresses. The treatment options included in the EDA are the combined oral contraceptive pill (COCP), contraceptive patch, vaginal ring, progestin-only pill (POP), non-contraceptive oral progestins, implant, injectable progestin, levonorgestrel intrauterine device (LNG-IUD), copper IUD, antifibrinolytics and non-steroidal anti-inflammatories (NSAIDs). The EDA was designed to be used during clinical encounters, with discussions oriented around the practical issues most relevant and important to the participants. We specifically designed this EDA to be accessible and clear to AYAs with DD or their caregiver, employing a low health literacy approach to ensure maximal comprehension. The cards were used to engage AYAs with DD as actively as possible capturing their preferences, concerns and needs. Caregiver's involvement was determined by the patient's preference and/or needs. In cases of severe impairment, the EDA was exclusively used by the caregiver.

### 2.2 Study design

We conducted a feasibility study to assess the acceptability of this EDA among AYAs with DD and their caregiver.

#### 2.2.1 Study setting

The study was conducted at the Pediatric and Adolescent Gynecology Consultation, within the Department of Pediatrics, Obstetrics and Gynecology at Geneva University Hospitals, Geneva, Switzerland. Consultations were conducted by three clinicians specialized in pediatric and adolescent gynecology.

### 2.3 Participants

Eligibility criteria included AYAs under the age of 26 years with DD who were post-menarchal and referred to the Pediatric and Adolescent Gynecology Consultation for discussion regarding menstrual management, as well as their caregivers. DD was defined as lifelong conditions associated with impairment in physical, cognitive, language and/or behavioral areas, with onset before the age of 22 [2,3]. Participants were excluded if neither the individual nor their caregiver were able to read and understand French.

#### 2.3.1 Recruitment

The principal investigator identified consecutive patients referred to the Pediatric and Adolescent Gynecology consultation. Both AYAs with DD and their caregivers were invited to participate in the study between May and August 2024.

#### 2.3.2 Outcomes and measures

The primary outcome was the acceptability of the decision aid, assessed using the French version of the Acceptability scale [38] immediately following the clinical encounter. This validated scale was developed by the Ottawa Decision Support Framework. The Acceptability scale does not yield a total score; instead, individualized items are analyzed separately. Question 3 was removed because it was deemed inappropriate for the setting, and responses to question 4 were modified to align with the context. The scale includes three questions with free-text responses.

Secondary outcomes included treatment preferences, decisional conflict and preparation for decision-making. We measured decisional conflict before and after the use of the EDA using the French version of the Decision Conflict Scale (DCS) [39]. The DCS is a 16-item validated scale that has been developed by the Ottawa Decision Support Framework. A total score is calculated and standardized to a scale ranging from 0 (no decisional conflict) to 100 (extremely high decisional conflict), with scores above 37.5 indicating high decisional conflict. We assessed preparation for decision making using the French version of the Preparation for Decision Making Scale (PDMS) [40], also developed and validated by the Ottawa Decision Support Framework. The PDMS provides a total score standardized to a 0–100 scale, where higher scores reflect a greater perceived level of preparedness for decision-making.

Health and disability level was measured using the 12-item WHODAS 2.0 [41], which evaluates functioning across six domains of life. Percentage scoring was calculated following the template provided by WHO, yielding a total score ranging from 0 (no limitation in functioning) to 100 (severe limitation in functioning).

We assessed mental well-being using the WHO-5 [42]. Percentage scoring generated a total score ranging from 0 (poor) to 100 (excellent).

We collected socio-demographic data, including age, developmental disability diagnosis and associated comorbidities, functional limitations, sexual activity, weight, and height, from the medical record. Menstrual characteristics such as age at menarche, menstrual regularity and length, bleeding volume, associated pain, behavioral changes, seizures, impact on school/sport participation, and any previous treatments used were also documented.

#### 2.3.3 Statistical analyses

Statistical analyses were performed using Stata 17.0 [43]. Due to the nature of this feasibility study, descriptive statistics were performed, with categorical data reported as numbers and percentages and continuous data as median and interquartile range (IQR) because of their skewed distribution. Considering the preliminary nature of this study, a paired sample Wilcoxon text was performed to test the difference between decisional conflict before and after the use of the EDA. We highlight that we considered the difference in decisional conflict before and after the consultation as an exploratory secondary outcome, and caution should be applied when interpreting the results. We estimated the sample size of 15–20 participants, based on recommendations of 10–30 participants for pilot studies [44,45] or 10 % of the sample projected for the larger parent study [46]).

#### 2.3.4 Ethics

This study was deemed to fall outside the scope of Swiss legislation regulating research on human subjects, and therefore, approval from the competent local ethics committee was waived (Req-2024-00265). We obtained written informed consent from all participants. For the participants who were capable of judgement, but unable to provide informed consent, their assent was obtained along with the consent of their legal representative. For the participants deemed incapable of judgement, consent was obtained exclusively by their legal representative.

## 3 Results

We identified 18 patients who met the eligibility criteria during the study period and all of them were included (none declined participation).

Table 1 described the clinical characteristics of the AYAs with DD. The most common comorbidities were genetic syndromes (10/18, 55.6 %) and autism spectrum disorder (7/18, 38.9 %). Intellectual disability was present in most individuals (15/18, 83.3 %). The level of health and disability was significant, with a median WHODAS 2.0 of 52. Median mental well-being as measured by the WHO-5 was 62.

**Table 1:** Demographics.

	Total
Number of individuals, n (%)	18 (100 %)
Age, median (IQR) (years)	17.8 (15.4–19.7)
BMI, median (IQR) (kg/m2)	21.5 (18.9–24.3)
WHODAS 2.0, median (IQR)	52 (33–85)
WHO-5, median (IQR)	66 (48–88)
Comorbidities, n/N (%)^a^	
Genetic syndrome^b^	10/18 (55.6 %)
Autism Spectrum Disorder	7/18 (38.9 %)
Epilepsy	5/18 (27.8 %)
Cerebral palsy	1/18 (5.6 %)
Brain trauma	1/18 (5.6 %)
Foeto-alcoholic syndrome	1/18 (5.6 %)
Functional limitations, n/N (%)^a^	
Intellectual disability	15/18 (83.3 %)
Non verbal	6/18 (33.3 %)
Diaper users	6/18 (33.3 %)
Wheelchair users	3/18 (16.7 %)
Hearing impairment	1/18 (5.6 %)
Feeding tube	0/18 (0 %)

BMI = Body Mass Index.

a Total is higher than 18 because AYAs could have multiple comorbidities or functional limitations.

b Including: Angelman syndrome, Barakat syndrome, Distal 18q deletion syndrome, Guanidinoacetate methyltransferase deficiency, IQSEC2-related disorder, Mowat-Wilson syndrome, Phelan-McDermid syndrome, Rett-like syndrome, Verheij syndrome, 8p inverted duplication/deletion syndrome.

The median age at menarche was 12 years old (IQR 2), with a median gynecological age of 5.3 years at the time of the consultation (IQR 3.8). Table 2 summarized menstruation characteristics. Over half of the individuals required assistance with menstrual hygiene, and nearly a third reported absenteeism from school, work or social activities due to menstruation. Period pain was the most cited reason for seeking menstrual management (11/18, 61.1 %). Only a minority had previously used or were currently using hormonal treatment for menstrual management.

**Table 2:** : Menstruation characteristics.

	Total
Regular menstruation (21–45 days), n/N (%)	13/18 (72.2 %)
Duration of menstruation <7 days, n/N (%)	13/18 (72.2 %)
Menstrual flow, n/N (%)	
Mild	3/18 (16.7 %)
Moderate	12/18 (66.7 %)
Heavy	3/18 (16.7 %)
Pain with menstruation, n/N (%)	14/18 (77.8 %)
Need help for hygiene with menstruation, n/N (%)	10/18 (55.6 %)
Behavior issues with menstruation, n/N (%)	7/18 (38.9 %)
School/work or social absenteeism due to menstruation, n/N (%)	5/18 (27.8 %)
Catamenial epilepsy, n/N (%)	2/18 (11.1 %)
Main reason for menstrual management, n/N (%)	
Pain	11/18 (61.1 %)
Behavior issues	3/18 (16.7 %)
Contraception	2/18 (11.1 %)
Hygiene	1/18 (5.6 %)
Catamenial epilepsy	1/18 (5.6 %)
Hormonal treatment for menstrual management	
Current use of hormonal treatment for menstrual management, n/N (%)	4/18 (22.2 %)
Levonorgestrel intrauterine device	2/4 (50.0 %)
Combined oral contraceptive pill	1/4 (25.0 %)
Progestin only pill	1/4 (25.0 %)
Past use of hormonal treatment for menstrual management, n/N (%)	3/18 (16.7 %)
Combined oral contraceptive pill	2/3 (66.7 %)
Progestin only pill	1/3 (33.3 %)

Two AYAs reported consensual sexual intercourse, while one individual disclosed sexual abuse. Nevertheless, many caregivers were unable to provide information about sexual activity due to communication limitations with the patient.

The decision aid was used primarily by the caregiver alone in 12/18 (66.7 %), by the patient alone in 4/18 (22.2 %) and jointly by both in 2/18 (11.1 %).

The acceptability of the EDA was high (Table 3). Most participants rated the presentation of information as either “excellent” (12/18, 66.7 %) or “good” (5/18, 27.8 %). Additionally, the majority found the EDA to be “very useful” (15/18, 83.3 %) or “useful” (3/18, 16.7 %) in facilitating decision-making.

**Table 3:** Acceptability scale.

	Total
What do you think about the way the information was presented?	
Excellent	12/18 (66.7 %)
Good	5/18 (27.8 %)
Fair	1/18 (5.6 %)
Poor	0/18 (0 %)
What do you think about the amount of information?	
too little information	0/18 (0 %)
just right	18/18 (100.0 %)
too much information	0/18 (0 %)
Do you find the tool…?	
inclined towards a treatment compared to another	4/18 (22.2 %)
balanced	14/18 (77.8 %)
inclined towards treatments compared to doing nothing	0/18 (0 %)
Do you think the tool was...?	
very useful	15/18 (83.3 %)
useful	3/18 (16.7 %)
a little useful	0/18 (0 %)
unuseful	0/18 (0 %)
Do you think we included enough information to help someone decide on treatment options for menstrual management?	
yes	18/18 (100.0 %)
no	0/18 (0 %)

In response to the question “What did you like about this decision aid?”, participants highlighted the ease of understanding the information, the simplicity and clarity of the data presentation, and the use of visual support. Several participants appreciated the ability to compare treatment options side by side. One participant noted that the cards were playful. No participant responded to the question, “What did not you like about this decision aid?”. Two suggestions for improvement were made: one suggested adding a card about hirsutism and acne. Another indicated including more descriptive details about treatment initiation or implementation (for example, the need for a gynecological exam involving a speculum for IUD insertion).

Prior to the consultation, more than half of the participants (10/18, 55.6 %) were unsure of their preferred treatment option (Table 4). Among those with an initial preference, the COCP and the progestin injection were the most frequently preferred options. After using the EDA, only one participant (1/18, 5.6 %) remained undecided. The COCP emerged as the most commonly chosen method (7/18, 38.9 %), followed by the contraceptive patch, POP and the LNG IUD (each selected by 2/18, 11.1 %). Notably, three participants (3/18, 16.7 %) decided not to initiate any hormonal treatment, opting instead for NSAIDs and/or antifibrinolytics.

**Table 4:** Exploratory outcomes.

	Before consultation	After consultation
Preference of treatment, n/N (%)		
Combined oral contraceptive pill	3/18 (16.7 %)	7/18 (38.9 %)
Contraceptive patch	0/18 (0 %)	2/18 (11.1 %)
Progestin only pill	1/18 (5.6 %)	2/18 (11.1 %)
Progestin injection	2/18 (11.1 %)	0/18 (0 %)
Progestin implant	1/18 (5.6 %)	1/18 (5.6 %)
Levonorgestrel intrauterine device	1/18 (5.6 %)	2/18 (11.1 %)
Does not know	10/18 (55.6 %)	1/18 (5.6 %)
No hormonal treatment	0/18 (0 %)	3/18 (16.7 %)
DCS total score, median (IQR)	40.0 (33.0 to 67.0)	15.0 (6.0 to 27.0)
Informed subscale	54.0 (42.0 to 75.0)	4.0 (0.0 to 17.0)
Values clarification subscale	50.0 (33.0 to 83.0)	8.0 (0.0 to 25.0)
Support subscale	33.0 (17.0 to 50.0)	8.0 (0.0 to 17.0)
Uncertainty subscale	42.0 (25.0 to 67.0)	25.0 (8.0 to 33.0)
Effective decision subscale	N/A	19.0 (6.0 to 31.0)
Preparation for decision making total score, median (IQR)	N/A	93.0 (85.0 to 98.0)

DCS = decision conflict scale.

Before the consultation, decisional conflict was high (>37.5), as indicated by the DCS total score and its subscales, except the support subscale (Table 4). Following the use of the EDA, decisional conflict decreased substantially, with a total DCS total score and all its subscales reported low (< 37.5). Using a paired sample Wilcoxon text, DCS total scores were significantly lower after using the EDA (median = 15.0, IQR = 21) compared to before the consultation (median = 40, IQR = 34; z = −3.725, *p* < 0.001). Additionally, preparation for decision-making was reported to be high (Table 4).

## 4 Discussion and conclusion

### 4.1 Discussion

This feasibility study aimed to assess the acceptability of an encounter decision aid (EDA) among adolescents and young adults (AYAs) with developmental disabilities (DD) and their caregivers when considering treatment options for menstrual management.

Our findings indicate that the EDA was highly acceptable to participants. Both AYAs and their caregivers consistently reported that the information was clearly presented and helpful in facilitating decision-making. Participants particularly appreciated the side-by-side comparison of treatment options side by side, which fostered a dynamic, iterative decision-making process during the clinical encounter. This “back and forth” process typically involved participants identifying one or two preferred options initially, then refining or reconsidering their choices as they reviewed additional cards. This iterative approach often led to meaningful conversations between clinicians and patients, often prompting practical questions and, in some cases, the emergence of a tentative treatment plan from patients (e.g. “I will try with the patch and, if I get skin irritation, I will change for the pill”). Breslin et al. [30] described a similar think-aloud approach with the use of their respective EDA. From a clinician's perspective, this process provided a valuable opportunity to deliver tailored information addressing the patient's specific concerns, while simultaneously assessing the patient's understanding and capacity for decision-making.

Shared decision-making is a collaborative process in which patients and clinicians work together to make decisions about treatments or interventions, using the best available evidence and taking into account patient's values and preferences [16,17]. This approach is particularly suited for healthcare decisions involving multiple options with comparable effectiveness and where patient preferences play a pivotal role. Since the goal of decision aids is to encourage shared decision-making, decision aids are better deployed during the clinical encounter (as EDA) as they facilitate the collaboration between patients and clinicians to reach a decision that is both appropriate from a medical perspective and consistent with the individual's values and preferences.

We decided to adopt a paper format (as opposed to an electronic format) for the EDA guided by several considerations: the prototype was inexpensive to produce and easy to modify during the early stages of development. In addition, the physical format encouraged engagement by allowing patients and caregivers to handle the cards, fostering interaction and playfulness, as noted by one participant. Moreover, using cards influenced the dynamics of the medical encounter by altering “body language”; clinicians and patients were often physically closer or sitting side by side while reviewing the cards. This shift in physical positioning underscored the collaborative nature of the shared decision process, similarly observed by others [30].

One of the limitations of this study is that our EDA was primarily utilized by caregivers, accounting for two-thirds of cases. This is an important consideration, as caregivers and AYAs with DD may have different perspectives and feedback, and developing decision aids for each group may require distinct approaches. The high level of caregiver involvement in using the decision aid and participating in the study likely reflects the large proportion of AYAs with severe DD in our cohort. We designed the EDA to present information utilizing pictograms and minimal text to accommodate varying levels of functioning among AYAs with DD. Despite these adaptations, the degree of involvement by AYAs varied significantly depending on their abilities and decision-making capacities. AYAs with DD demonstrate a broad spectrum of cognitive and functional abilities. Some can independently decide on treatment options, such as choosing between a daily pill or a three-monthly injection for dysmenorrhea management. Others may express preferences or opinions but require caregiver input, while those with severe impairments may rely entirely on caregivers to make decisions on their behalf.

Shared decision-making hinges on a patient's ability to comprehend the decision, weigh the benefits and risks of available options, communicate their preferences. However, impaired decision-making capacity does not preclude participation in shared decision-making [47]. Decision-making capacity is highly personal and dynamic, varying by decision type and fluctuating over time. For example, an adolescent with mild or moderate cognitive impairment might be able to decide whether they want a daily pill or prefer a three-monthly injection for managing dysmenorrhea. While AYAs with DD are being entitled to privacy, autonomy, and confidentiality as their peers, involving caregivers in at least part of the clinical encounter may also be helpful or required to facilitate understanding or communication between the patient and the clinician. Some AYAs with DD may benefit from supported decision-making, in which an individual with impaired capacity willingly engages with a trusted person who assists in understanding and making decisions [48].

### 4.2 Innovation

Professional societies guidelines emphasize that gynecologists should engage in shared decision-making with their patients [11,49]. While several decision aids have been developed for contraceptive decision-making, for both reversible and irreversible methods, few were specifically designed for use during clinical encounters and their efficacy has shown inconsistent results [50,51]. Similarly, a small number of decision aids have been created for heavy menstrual bleeding, but none of them were intended for use within the consultation setting nor were they specifically tailored for adolescents [52]. This distinction is crucial as adolescents often face unique menstrual issues different from adults (e.g. worsening acne with menstrual cycle), and some interventions may be inappropriate for this population (e.g. hysterectomy for heavy menstrual bleeding).

Individuals with DD constitute a vulnerable population that continues to experience significant inequities in healthcare, including in menstrual and reproductive health services. Growing evidence indicates that AYAs with DD are more likely affected by menstrual disorders such as dysmenorrhea, amenorrhea, irregular bleeding and premenstrual syndrome compared to their healthy peers [53,54]. Nevertheless, AYAs with DD are less likely to visit a gynecologist and are less commonly prescribed hormonal medication for menstrual management compared to the general population [53]. This discrepancy reflects many barriers encountered by AYAs with DD, including insufficient training of healthcare providers in the care of individuals with disabilities, low provider confidence, poor communication skills, and discomfort in addressing sexual and reproductive health. Additional contributing factors include misconceptions that individuals with DD are not sexually active, and a lack of comprehensive sexual health education tailored to AYAs with DD.

To overcome some of these barriers, it has been suggested to adapt communication strategies when interacting with individuals with DD, ensuring information is conveyed in accessible formats and actively involving them in their care as much as possible [55,56]. Nevertheless, research on engaging individuals with DD in decision-making remains limited, and few decision aids have been developed for this population [[20], [21], [22]]. To the best of our knowledge, this is the first decision aid specifically developed to address treatment options for menstrual management in AYAs with DD. This innovative tool may support shared decision-making for AYAs with DD and their caregivers, and provide additional insight on how to engage individuals with DD in healthcare decisions. Further research should explore strategies to enhance shared decision-making for AYAs with DD, focusing on increasing their involvement in healthcare decisions while respecting their unique abilities and needs.

## 5 Conclusion

In conclusion, we developed an EDA named “Changeons les règles!” to support clinicians in facilitating conversation about treatment options for menstrual management and to encourage AYAs with DD and their caregivers to engage in shared decision-making. This EDA is both evidence-based and user-centered. Further research is needed to determine its effectiveness in promoting shared decision-making in this vulnerable population, and its long-term impact on patient satisfaction and well-being.

## Ethics approval and consent to participate

This study was considered as falling outside of the scope of the Swiss legislation regulating research on human subjects, so that the need for competent local ethics committee approval was waived (Req-2024-00265). Written informed consent of all participants was obtained. In the event of a participant capable of judgement but not of consent, their assent was collected in addition to the consent of their legal representative. In the event of a participant incapable of judgement, the consent of their legal representative was collected.

## Consent for publication

Not applicable.

## Availability of data and materials

The datasets used and/or analyzed during the current study are available from the corresponding author on reasonable request.

## Funding

Dehlia Moussaoui is supported by a grant “cheffe de clinique scientifique” from the 10.13039/501100006389University of Geneva, Geneva, Switzerland.

Dehlia Moussaoui and Michal Yaron are supported by a grant from the “Fondation privée”, 10.13039/501100006388Geneva University Hospitals, Geneva, Switzerland.

The development team won the first prize for this tool development at the 7th edition of Hackathon, organized by the “Centre de l'Innovation des HUG”.

## CRediT authorship contribution statement

**Dehlia Moussaoui:** Writing – review & editing, Writing – original draft, Resources, Methodology, Investigation, Formal analysis, Data curation, Conceptualization. **Victoria L. Crofts:** Writing – review & editing, Formal analysis, Data curation, Conceptualization. **Anne-Chantal Héritier-Barras:** Writing – review & editing, Supervision, Resources, Conceptualization. **Thomas Agoritsas:** Writing – review & editing, Validation, Supervision, Formal analysis. **Arnaud Merglen:** Writing – review & editing, Validation, Supervision, Formal analysis, Conceptualization. **Michal Yaron:** Writing – review & editing, Validation, Supervision, Formal analysis, Conceptualization.

## Declaration of competing interest

The authors declare the following financial interests/personal relationships which may be considered as potential competing interests:

Dehlia Moussaoui, Victoria Crofts and Michal Yaron reports financial support was provided by Private Foundation HUG. Dehlia Moussaoui reports financial support was provided by University of Geneva. If there are other authors, they declare that they have no known competing financial interests or personal relationships that could have appeared to influence the work reported in this paper.

## Appendix A Supplementary data

Supplementary material Image 1

## References

[1] Munro C.B., Walker E.N., Schembri R., Moussaoui D., Grover S.R. (2023). Periods shouldn't bring any adolescents' world to a full stop. Period An online survey of adolescents' experience of menstruation. J Pediatr Adolesc Gynecol.

[2] Brown I., Wehmeyer M.L., Shogren K.A., Wehmeyer M.L., Brown I., Percy M., Shogren K.A., Fung W.L.A. (2017). A comprehensive guide to intellectual and developmental disabilities.

[3] CDC (2025). Developmental Disability Basics. https://www.cdc.gov/child-development/about/developmental-disability-basics.html#:∼:text=Developmental%20disabilities%20are%20a%20group,last%20throughout%20a%20person's%20lifetime.

[4] Steward R., Crane L., Roy E.M., Remington A., Pellicano E. (2018). “Life is much more difficult to manage during periods”: autistic experiences of menstruation. J Autism Dev Disord.

[5] Frances Fei Y., Ernst S.D., Dendrinos M.L., Quint E.H. (2021). Satisfaction with hormonal treatment for menstrual suppression in adolescents and young women with disabilities. J Adolesc Health.

[6] Jones L., Bellis M.A., Wood S., Hughes K., McCoy E., Eckley L. (2012). Prevalence and risk of violence against children with disabilities: a systematic review and meta-analysis of observational studies. Lancet.

[7] Basile K.C., Breiding M.J., Smith S.G. (2016). Disability and risk of recent sexual violence in the United States. Am J Public Health.

[8] Cummins C., Pellicano E., Crane L. (2020). Supporting minimally verbal autistic girls with intellectual disabilities through puberty: perspectives of parents and educators. J Autism Dev Disord.

[9] Powell R.M., Parish S.L., Mitra M., Rosenthal E. (2020). Role of family caregivers regarding sexual and reproductive health for women and girls with intellectual disability: a scoping review. J Intellect Disabil Res.

[10] Quint E.H., O’Brien R.F., COMMITTEE ON ADOLESCENCE, The North American Society for Pediatric and Adolescent Gynecology, Braverman P.K., Adelman W.P., Alderman E.M. (2016). Pediatrics.

[11] American College of Obstetricians and Gynecologists’’ Committee on Clinical Consensus–Gynecology (2022). General Approaches to Medical Management of Menstrual Suppression: ACOG Clinical Consensus No. 3. Obstet Gynecol.

[12] Flavin M., Shore B.J., Miller P., Gray S. (2019). Hormonal contraceptive prescription in young women with cerebral palsy. J Adolesc Health.

[13] Acharya K., Lantos J.D. (2016). Considering decision-making and sexuality in menstrual suppression of teens and young adults with intellectual disabilities. AMA J Ethics.

[14] Stacey D., Lewis K.B., Smith M., Carley M., Volk R., Douglas E.E. (2024). Decision aids for people facing health treatment or screening decisions. Cochrane Database Syst Rev.

[15] Feldman-Stewart D., O’Brien M.A., Clayman M.L., Davison B.J., Jimbo M., Labrecque M. (2013). Providing information about options in patient decision aids. BMC Med Inform Decis Mak.

[16] Stiggelbout A.M., Van der Weijden T., De Wit M.P.T., Frosch D., Légaré F., Montori V.M. (2012). Shared decision making: really putting patients at the Centre of healthcare. BMJ.

[17] Elwyn G., Lloyd A., May C., van der Weijden T., Stiggelbout A., Edwards A. (2014). Collaborative deliberation: a model for patient care. Patient Educ Couns.

[18] Heen A.F., Vandvik P.O., Brandt L., Montori V.M., Lytvyn L., Guyatt G. (2021). A framework for practical issues was developed to inform shared decision-making tools and clinical guidelines. J Clin Epidemiol.

[19] Heen A.F., Vandvik P.O., Brandt L., Achille F., Guyatt G.H., Akl E.A. (2021). Decision aids linked to evidence summaries and clinical practice guidelines: results from user-testing in clinical encounters. BMC Med Inform Decis Mak.

[20] Noorlandt H.W., Korfage I.J., Felet F.M.A.J., Aarts K., Festen D.A.M., Vrijmoeth C. (2024). Shared decision making with frail people with intellectual disabilities in the palliative phase: a process evaluation of the use of the In-Dialogue conversation aid in practice. Research Intellect Disabil.

[21] Fisher Z., Bailey R., Willner P. (2012). Practical aspects of a visual aid to decision making. J Intellect Disabil Res.

[22] Bailey R., Willner P., Dymond S. (2011). A visual aid to decision-making for people with intellectual disabilities. Res Dev Disabil.

[23] O'Connor A.M. (1998). Development methods for Ottawa patient decision aids. https://decisionaid.ohri.ca/methods.html.

[24] Coulter A., Stilwell D., Kryworuchko J., Mullen P.D., Ng C.J., van der Weijden T. (2013). A systematic development process for patient decision aids. BMC Med Inform Decis Mak.

[25] Moussaoui D., Laqua J., Crofts V.L., Nemitz-Piguet C.M., Héritier-Barras A.-C., Merglen A. (2025). Menstrual management using hormonal medications in adolescents and young adults with developmental disability: a systematic review and a meta-analysis. J Obstet Gynaecol.

[26] American College of Obstetricians and Gynecologists’’ cCommittee on aAdolescent hHealth cCare (2016). committee opinion No. 668: Menstrual manipulation for adolescents with physical and developmental disabilities. Obstet Gynecol.

[27] Gynecologic Management of Adolescents and Young Women With Seizure Disorders: ACOG Committee Opinion (2020). Number 806. Obstet Gynecol.

[28] Kirkham Y.A., Ornstein M.P., Aggarwal A., McQuillan S., No. (2019). 313-menstrual suppression in special circumstances. J Obstet Gynaecol Can.

[29] Montori V.M., Breslin M., Maleska M., Weymiller A.J. (2007). Creating a conversation: insights from the development of a decision aid. PLoS Med.

[30] Breslin M., Mullan R.J., Montori V.M. (2008). The design of a decision aid about diabetes medications for use during the consultation with patients with type 2 diabetes. Patient Educ Couns.

[31] Hess E.P., Hollander J.E., Schaffer J.T., Kline J.A., Torres C.A., Diercks D.B. (2016). Shared decision making in patients with low risk chest pain: prospective randomized pragmatic trial. BMJ.

[32] Mullan R.J., Montori V.M., Shah N.D., Christianson T.J.H., Bryant S.C., Guyatt G.H. (2009). The diabetes mellitus medication choice decision aid: a randomized trial. Arch Intern Med.

[33] LeBlanc A., Herrin J., Williams M.D., Inselman J.W., Branda M.E., Shah N.D. (2015). Shared decision making for antidepressants in primary care: a cluster randomized trial. JAMA Intern Med.

[34] Kunneman M., Branda M.E., Hargraves I.G., Sivly A.L., Lee A.T., Gorr H. (2020). Shared decision making for atrial fibrillation (SDM4AFib) trial investigators, assessment of shared decision-making for stroke prevention in patients with atrial fibrillation: a randomized clinical trial. JAMA Intern Med.

[35] Mann D.M., Ponieman D., Montori V.M., Arciniega J., McGinn T. (2010). The statin choice decision aid in primary care: a randomized trial. Patient Educ Couns.

[36] Montori V.M., Shah N.D., Pencille L.J., Branda M.E., Van Houten H.K., Swiglo B.A. (2011). Use of a decision aid to improve treatment decisions in osteoporosis: the osteoporosis choice randomized trial. Am J Med.

[37] Hess E.P., Homme J.L., Kharbanda A.B., Tzimenatos L., Louie J.P., Cohen D.M. (2018). Effect of the head computed tomography choice decision aid in parents of children with minor head trauma: a cluster randomized trial. JAMA Netw Open.

[38] O’Connor A.M., Cranney A. (2025). http://decisionaid.ohri.ca/docs/develop/User_Manuals/UM_Acceptability.pdf.

[39] O’Connor A.M. (2015). https://decisionaid.ohri.ca/docs/develop/User_Manuals/UM_Decisional_Conflict.pdf.

[40] Graham I.D., O’Connor A.M. (2025). https://decisionaid.ohri.ca/docs/develop/User_Manuals/UM_PrepDM.pdf.

[41] Ustün T.B., Chatterji S., Kostanjsek N., Rehm J., Kennedy C., Epping-Jordan J. (2010). WHO/NIH joint project, developing the World Health Organization disability assessment schedule 2.0. Bull World Health Organ.

[42] Topp C.W., Østergaard S.D., Søndergaard S., Bech P. (2015). The WHO-5 well-being index: a systematic review of the literature. Psychother Psychosom.

[43] StataCorp (2021).

[44] Isaac S., Michael W. (1995).

[45] Julious S.A. (2005). Sample size of 12 per group rule of thumb for a pilot study. Pharm Stat.

[46] Connelly L.M. (2008). Pilot studies. Medsurg Nurs.

[47] Lahey T., Elwyn G. (2020). Sliding-scale shared decision making for patients with reduced capacity. AMA J Ethics.

[48] Peterson A., Karlawish J., Largent E. (2021). Supported decision making with people at the margins of autonomy. Am J Bioeth.

[49] American College of Obstetricians and Gynecologists'’ Committee on Health Care for Underserved Women (2022). Contraceptive equity expert work group, and committee on ethics, patient-centered contraceptive counseling: acog committee statement number 1. Obstet Gynecol.

[50] Goueth R.C., Maki K.G., Babatunde A., Eden K.B., Darney B.G. (2022). Effects of technology-based contraceptive decision aids: a systematic review and meta-analysis. Am J Obstet Gynecol.

[51] Jones A., Allison B.A., Perry M. (2022). Effectiveness of contraceptive decision aids in adolescents and young adults: a systematic review. J Pediatr Adolesc Gynecol.

[52] Zandstra D., Busser J.A.S., Aarts J.W.M., Nieboer T.E. (2017). Interventions to support shared decision-making for women with heavy menstrual bleeding: a systematic review. Eur J Obstet Gynecol Reprod Biol.

[53] Ames J.L., Anderson M.C., Cronbach E., Lee C., Onaiwu M.G., Vallerie A.M. (2024). Reproductive healthcare in adolescents with autism and other developmental disabilities. Am J Obstet Gynecol.

[54] Hamilton A., Marshal M.P., Murray P.J. (2011). Autism spectrum disorders and menstruation. J Adolesc Health.

[55] Barbera J.P., Cichon B., Ankam N., Schwartz B.I. (2024). Equitable Care for Patients with Disabilities: considerations for the gynecologic health care professional. Obstet Gynecol.

[56] Roden R.C., Schmidt E.K., Holland-Hall C. (2020). Sexual health education for adolescents and young adults with intellectual and developmental disabilities: recommendations for accessible sexual and reproductive health information. Lancet Child Adolesc Health.

