# Supplementary material for: “Changeons les Règles!” development and feasibility testing of an encounter decision aid for menstrual management in adolescents and young adults with developmental disabilities
Source: PEC Innov. 2025 Sep 13;7:100430. doi: 10.1016/j.pecinn.2025.100430 (PMC12482337; doi:10.1016/j.pecinn.2025.100430)
Supplement: Supplementary file 1 — Supplementary material [file mmc1.docx]

***PATIENT EDUCATION AND COUNSELING***

**CHECKLIST for the preparation of papers**

Please **ensure** that your paper conforms to the following guidelines:

**SPELLING**

In the manuscript either US or UK usage should be followed but not a mixture of these.

**AFFILIATIONS**

Forenames for all authors in the author list; no titles like Prof., Dr., etc.

Affiliations must appear in English

In the affiliations use USA and UK instead of United States and United Kingdom

The following should be followed for all authors’ affiliations: only Department,

University, City and Country

Provide full correspondence details (address including country, telephone, fax and e-mail address) for the corresponding author listed separately using the wording “Corresponding author at”

**ABSTRACT**

Abstracts should be structured and have the following headings:

Objectives; Methods; Results; Conclusions; Practice implications

**MAIN TEXT**

All Original Articles must contain a first order heading section “Discussion and

Conclusion” and three second order headings: (1) Discussion, (2) Conclusion, (3) Practice implications. Sub-headings are only permitted within the Discussion.

**DECLARATION OF COMPETING INTERESTS**

All articles must include a separate file containing a statement declaring any competing interests that relate to any authors. The below is the format. Please click either of the two options and submit the form.

☐ The authors declare that they have no known competing financial interests or personal relationships that could have appeared to influence the work reported in this paper.

☐The authors declare the following financial interests/personal relationships which may be considered as potential competing interests:

**REFERENCES**

Abbreviations for the names of journals in the reference list should follow Index

Medicus (e.g. JAMA should read J Amer Med Assoc, BMJ should read Brit Med J). The abbreviation for Patient Education and Counseling is Patient Educ Couns

Issue numbers and months must not be included in the reference list (only year, volume numbers and page range are required)

Page ranges in the reference list should appear as follows, e.g. 310-5

Titles of non English publications should be given in English language, between [ ]

**For further details see the extended guidelines**
